# Liver X Receptor ligand cytotoxicity in colon cancer cells and not in normal colon epithelial cells depends on LXRβ subcellular localization

**DOI:** 10.18632/oncotarget.5791

**Published:** 2015-09-22

**Authors:** Flavie Courtaut, Valentin Derangère, Angélique Chevriaux, Sylvain Ladoire, Alexia K. Cotte, Laurent Arnould, Romain Boidot, Mickaël Rialland, François Ghiringhelli, Cédric Rébé

**Affiliations:** ^1^ Institut National de la Santé et de la Recherche Médicale (INSERM) UMR 866, Dijon, France; ^2^ UFR des sciences de santé, Université de Bourgogne, Dijon, France; ^3^ Centre Georges François Leclerc, Dijon, France; ^4^ Faculté des sciences, Université de Bourgogne, Dijon, France

**Keywords:** LXRβ, RXRα, colon cancer, epithelial cells, subcellular localization

## Abstract

Increasing evidence indicates that Liver X Receptors (LXRs) have some anticancer properties. We recently demonstrated that LXR ligands induce colon cancer cell pyroptosis through an LXRβ-dependent pathway. In the present study, we showed that human colon cancer cell lines presented differential cytoplasmic localizations of LXRβ. This localization correlated with caspase-1 activation and cell death induction under treatment with LXR ligand. The association of LXRβ with the truncated form of RXRα (t-RXRα) was responsible for the sequestration of LXRβ in the cytoplasm in colon cancer cells. Moreover t-RXRα was not expressed in normal colon epithelial cells. These cells presented a predominantly nuclear localization of LXRβ and were resistant to LXR ligand cytotoxicity.

Our results showed that predominant cytoplasmic localization of LXRβ, which occurs in colon cancer cells but not in normal colon epithelial cells, allowed LXR ligand-induced pyroptosis. This study strengthens the hypothesis that LXRβ could be a promising target in cancer therapy.

## INTRODUCTION

In addition to their role in lipid metabolism, the nuclear receptors Liver X Receptor α (LXRα or NR1H3) and β (or NR1H2) have been shown to control cancer cell proliferation and to a lesser extent, cancer cell death, *in vitro* and *in vivo,* [1]. A common feature of these reports is that all these mechanisms seem to involve only the transcriptional activity of LXRs. On the other hand, we and others have reported that LXRs can also induce cancer cell death [1]. These effects may be induced by the transcriptional activation of LXR target genes implicated in lipid metabolism. The induction of ABCG1 expression leads to membrane lipid raft disruption, the inhibition of serine/threonine protein kinase Akt activity and caspase activation in prostate cancer cells. The induction of IDOL (Inducible degrader of the LDLR (Low Density Lipoprotein Receptor)) expression drives LDLR degradation in glioblastoma cells, which in turn induces cell death [2, 3]. We recently demonstrated that LXR agonists can induce colon cancer cell death independently of any transcriptional activity. In particular, the first molecular events that eventually leads to cell death occur within the first minutes of treatment and consist of ATP release in the supernatant of the cells through the pannexin 1 channel. Then ATP acts on its receptor P2×7 to trigger NLRP3 (Nod-Like-Receptor Pyrin domain containing 3) inflammasome-mediated caspase-1 activation. Finally caspase-1 induces cell death by pyroptosis [4, 5].

LXRs were previously reported to be localized in the nucleus of cells overexpressing fluorescent-tagged LXRα or LXRβ, in an NLS (Nuclear Localization Signal)-dependent manner [6, 7]. However, in the HCT116 colon cancer cell line, we reported that LXRβ was located in the cytoplasm rather than the nucleus [4]. The aim of this work was to study this atypical localization of LXRβ. We focused on the molecular mechanism responsible and on the possible correlation with colon cancer cell sensitivity to LXR agonist-mediated cell death. We demonstrated here that t-RXRα, the truncated form of RXRα (Retinoid X Receptor α), sequestrates LXRβ in the cytoplasm of colon cancer cells, thus potentiating the cytotoxic effects of agonist treatment. In contrast, because t-RXRα is absent from normal human colon epithelial cells, LXRβ is mainly located in the nucleus, thus diminishing the sensitivity of these cells to LXR ligand cytotoxicity.

## RESULTS

### Colon cancer cell lines show varying degrees of sensitivity to LXR agonist-induced cell death

We first tested the cytotoxic effects of the LXR agonist T0901317 on seven human colon cancer cell lines (HCT116, HT29, HCT8, SW480, SW620, LoVo and SW48). For this purpose, cells were treated for 72 hours with a range of T0901317 concentrations from 0 to 50 μM and cell viability was determined by crystal violet staining. From these results, EC50 (50% Efficacy concentrations) were calculated (Table 1). EC50 ranged from about 24 to 40μM, thus showing the different sensitivity of these cell lines to T0901317-mediated cytotoxicity. Some cell lines, such as HCT116 and HT29, presented a lower EC50, thus demonstrating higher sensitivity while others, such as SW620 and SW48, presented a higher EC50, thus demonstrating lower sensitivity. Similar results were obtained with FLICA-1 positive cells, which also accounts for the effects of T0901317 (Table 1). These results show the varying degrees of sensitivity of colon cancer cells to T0901317-induced cell death and caspase-1 activation.

**Table 1:** EC50 calculated after a treatment with a range of T0901317 concentrations for 72 hours

Cell lines	EC50 (μM) +/− s.d.	FLICA-1% cells (%) +/− s.d.
HCT116	24.2 +/− 1.5	13.7 +/− 2.5
HT29	25.8 +/− 0.5	10.0 +/− 1.2
HCT8	30.3 +/− 2.6	6.1 +/− 1.7
SW480	32.5 +/− 1.5	8.3 +/− 2.6
SW620	37.8 +/− 1.7	4.4 +/− 2.0
LoVo	28.2 +/− 0.2	8.5 +/− 3.1
SW48	39.9 +/− 5.6	4.6 +/− 1.7

FLICA-1 positive cells determined after 1 hour of treatment with 20μM of T0901317

### Colon cancer cell sensitivity does not correlate with LXRβ expression

**Figure 1:**
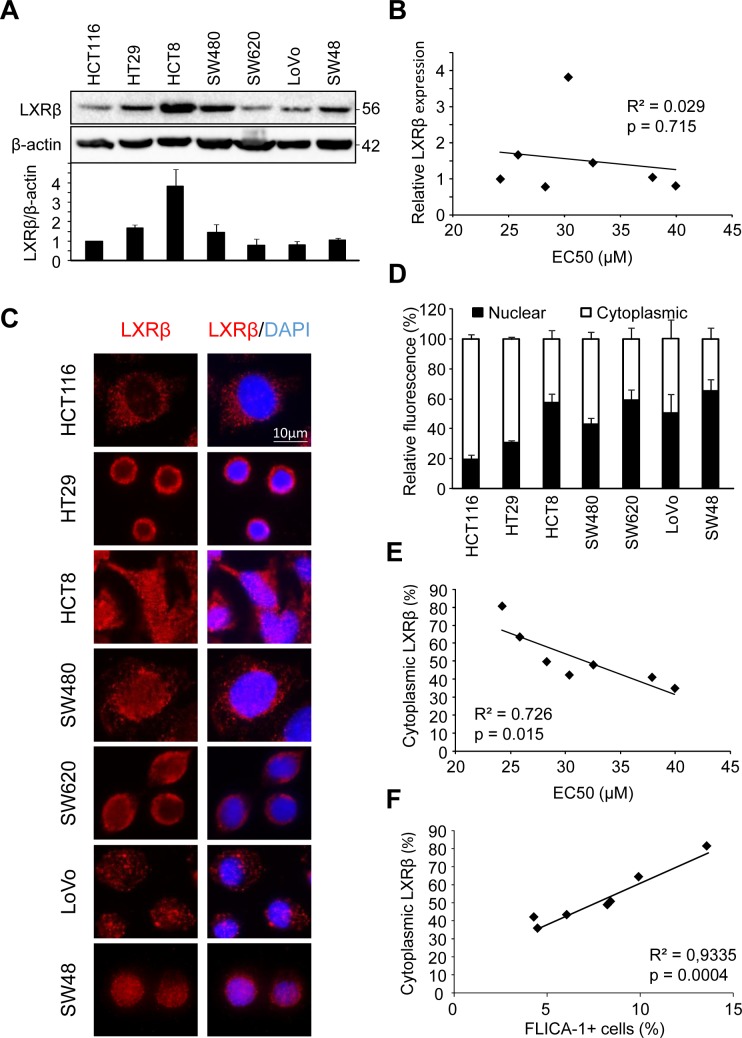
Human colon cancer cell sensitivity correlates with LXRβ localization **A.** Western blot analysis of LXRβ protein expression in HCT116, HT29, HCT8, SW480, SW620, LoVo and SW48 human colon cancer cell lines. β-Actin was used as a loading control. Numbers indicate molecular masses in kilodaltons. Upper panel: one representative experiment. Lower panel: mean of the quantification of the LXRβ/β-actin ratio in three different experiments ± s.d.. **B.** Relationship between LXRβ relative expression and EC50 (μM) calculated in table 1 in human colon cancer cell lines. Solid line represents linear regression curve. The regression coefficient (R^2^) and the Pearson correlation (p) are given. **C.** Representative images of immunofluorescence staining of LXRβ in colon cancer cells. Left: anti-LXRβ (red). Right: merge of LXRβ staining with DAPI (blue). **D.** Mean relative quantification of LXRβ fluorescence in the nucleus (black) and the cytoplasm (white) of colon cancer cells in three different experiments ± s.d.. (**E.** and **F.**) Relationship between LXRβ cytoplasmic distribution (%) and EC50 (μM) **E.** or FLICA-1 (%) **F.** calculated in table 1 in human colon cancer cell lines. Solid line represents linear regression curve. The regression coefficient (R^2^) and the Pearson correlation (p) are given.

In order to explain these variations in colon cancer cell response, we first checked whether there was differential expression of LXRβ (the isoform that we identified as responsible for T0901317-induced cell death in colon cancer cells [4]) by western blot (Figure 1A). Even though we noticed a difference in LXRβ expression between the tested cell lines, we found no correlation with the cell's sensitivity to T0901317-induced cell death (*p* > 0.05) (Figure 1B).

### Colon cancer cell sensitivity correlates with the subcellular localization of LXRβ

We therefore decided to explore the subcellular localization of LXRβ by immunofluorescence in the different cell lines used above (Figure 1C). Some cell lines, such as HCT116 and HT29, showed a predominantly cytoplasmic distribution of LXRβ (with about 80% of cytoplasmic LXRβ) while others, such as SW620 and SW48, presented a low cytoplasmic localization (with about 40%) (Figure 1D). These results were confirmed by generating nuclear and cytosolic fractions in HCT116 and SW480 cells (Supplementary Figure 1). Finally, we also noticed a statistically relevant correlation between colon cancer cell sensitivity and LXRβ cytoplasmic localization (*p* < 0.05) (Figure 1E and 1F). These results therefore show that the subcellular localization of LXRβ seems to be important in the response of colon cancer cell to treatment with an LXR agonist.

### LXRβ cytoplasmic localization correlates with t-RXRα expression

**Figure 2:**
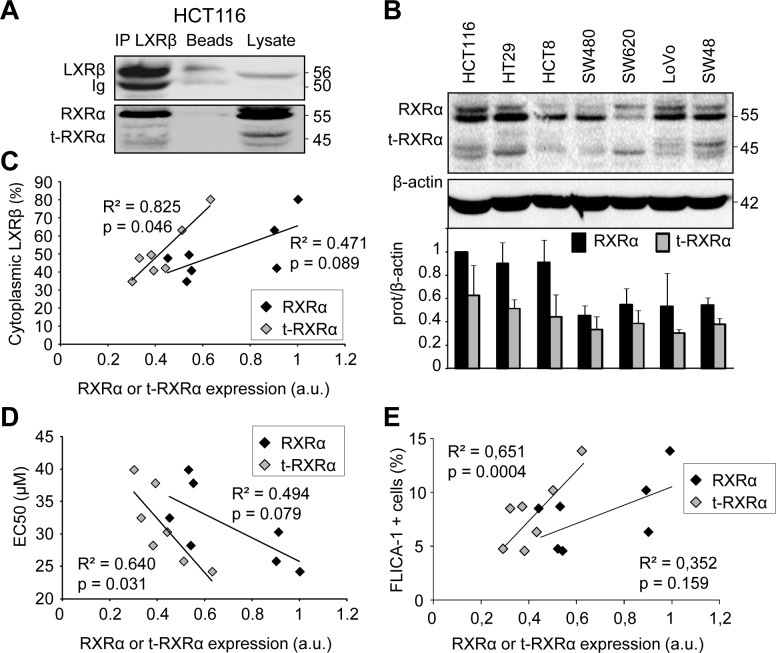
Human colon cancer cell sensitivity and LXRβ localization are correlated with t-RXRα expression **A.** Immunoprecipitation using LXRβ antibody on HCT116 cells and followed by western blot analysis using anti-LXRβ and anti-RXRα antibodies. Beads as negative controls without antibody for IP and total lysate were also loaded. Numbers indicate molecular masses in kilodaltons. One representative experiment out of three. **B.** Western blot analysis of RXRα protein expression in human colon cancer cell lines. β-Actin was used as a loading control. Numbers indicate molecular masses in kilodaltons. Upper panel: one representative experiment. Lower panel: mean quantification of the RXRα/β-actin (black) and the t-RXRα/β-actin (grey) ratios in three different experiments ± s.d.. Relationship between proportion of LXRβ in the cytoplasm (%) and relative expression of RXRα (black diamonds) or t-RXRα (grey diamonds) in human colon cancer cell lines. **C.** Relationship between EC50 (μM) and relative expression of RXRα (black diamonds) or t-RXRα (grey diamonds) in human colon cancer cell lines. **D.** Relationship between FLICA-1 positive cells and relative expression of RXRα (black diamonds) or t-RXRα (grey diamonds) in human colon cancer cell lines. **E.** Solid lines represent linear regression curves. The regression coefficient (R^2^) and the Pearson correlation (p) are given.

In order to explain the cytoplasmic LXRβ localization which was associated with sensitivity to T0901317-induced cell death, we first checked whether it was bound to its favorite partner RXRα [8]. After immunoprecipitating endogenous LXRβ in HCT116 cells we observed an interaction not only with RXRα, but also with its truncated form, t-RXRα (Figure 2A). This interaction still occurred after treatment with LXR or RXR agonists (not shown). The truncated RXRα has been described to result from a type II calpain cleavage of the N-terminal domain of RXRα specifically in cancer cells [9]. Moreover we showed that t-RXRα is mainly localized in the cytoplasm of colon cancer cells (Supplementary Figure 1). We observed a dissimilar expression of RXRα and t-RXRα in whole cell lysates obtained from the different cell lines tested (Figure 2B). While there was no statistically relevant correlation between LXRβ cytoplasmic localization and RXRα expression (*p* > 0.05), we observed a correlation between LXRβ cytoplasmic localization and t-RXRα expression (*p* < 0.05) (Figure 2C). Likewise, we only found a statistically relevant correlation between colon cancer cell sensitivity to T0901317 and t-RXRα expression (*p* < 0.05) but not with RXRα expression (*p* > 0.05) (Figure 2D and 2E). Overall, these results suggest that t-RXRα can control the subcellular localization of LXRβ and thus T0901317-induced cell death of colon cancer cells.

### RXRα/t-RXRα dictates LXRβ subcellular localization and colon cancer cell sensitivity to LXR ligand cytotoxicity

**Figure 3:**
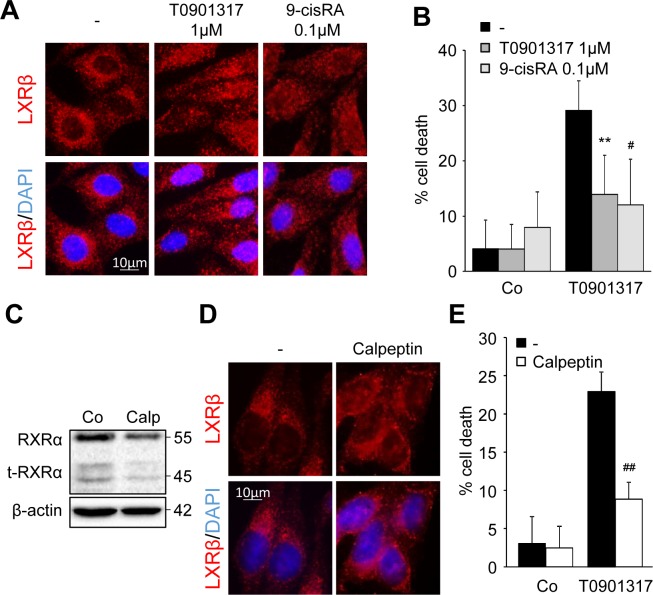
LXRβ nuclear relocalization decreases LXR ligand cytotoxicity HCT116 cells were pre-treated with either 1μM T0901317 or 0.1μM 9-cis RA (6h) **A.** - **B.** or with 10μM calpeptin (2h) **C.** - **E.** or vehicles (−). **A.** Immunofluorescence staining of LXRβ. Above: anti-LXRβ (red). Below: merge of LXRβ staining with DAPI (blue). **B.** Cell death determined by crystal violet coloration of cells pre-treated as indicated and exposed to 20μM of T0901317 for 24 hours. Mean of three independent experiments ± s.d.. **C.** Western blot analysis of RXRα and t-RXRα protein expression. β-Actin was used as a loading control. Numbers indicate molecular masses in kilodaltons. One representative experiment out of three. **D.** Immunofluorescence staining of LXRβ. Above: anti-LXRβ (red). Below: merge of LXRβ staining with DAPI (blue). **E.** Cell death determined by crystal violet coloration of cells pre-treated as indicated and exposed to 20μM of T0901317 for 24 hours. Mean of three independent experiments ± s.d.. Statistics compare cells treated only with T0901317 with other treated conditions: ^#^*p* < 0.01, **p < 0.005, ^##^*p* < 0.001, using two tailed t test.

Exposure of HCT116 cells to a low dose of the LXR ligand T0901317 (1μM) or to the well-known RXRα ligand, 9-cis Retinoic Acid (RA - 0.1μM) induced a relocalization of LXRβ to the nucleus (Figure 3A) without inducing caspase-1 activation (supplementary Figure 2 and data not shown). This pre-treatment also made the cells resistant to T0901317 cytotoxicity (Figure 3B). As previously shown, the inhibition of type II calpains with calpeptin reduced RXRα cleavage into t-RXRα (Figure 3C) [10]. This inhibition of t-RXRα production led to the redistribution of LXRβ into the nucleus (Figure 3D) and reduced the sensitivity of HCT116 to T0901317-mediated cytotoxicity (Figure 3E).

**Figure 4:**
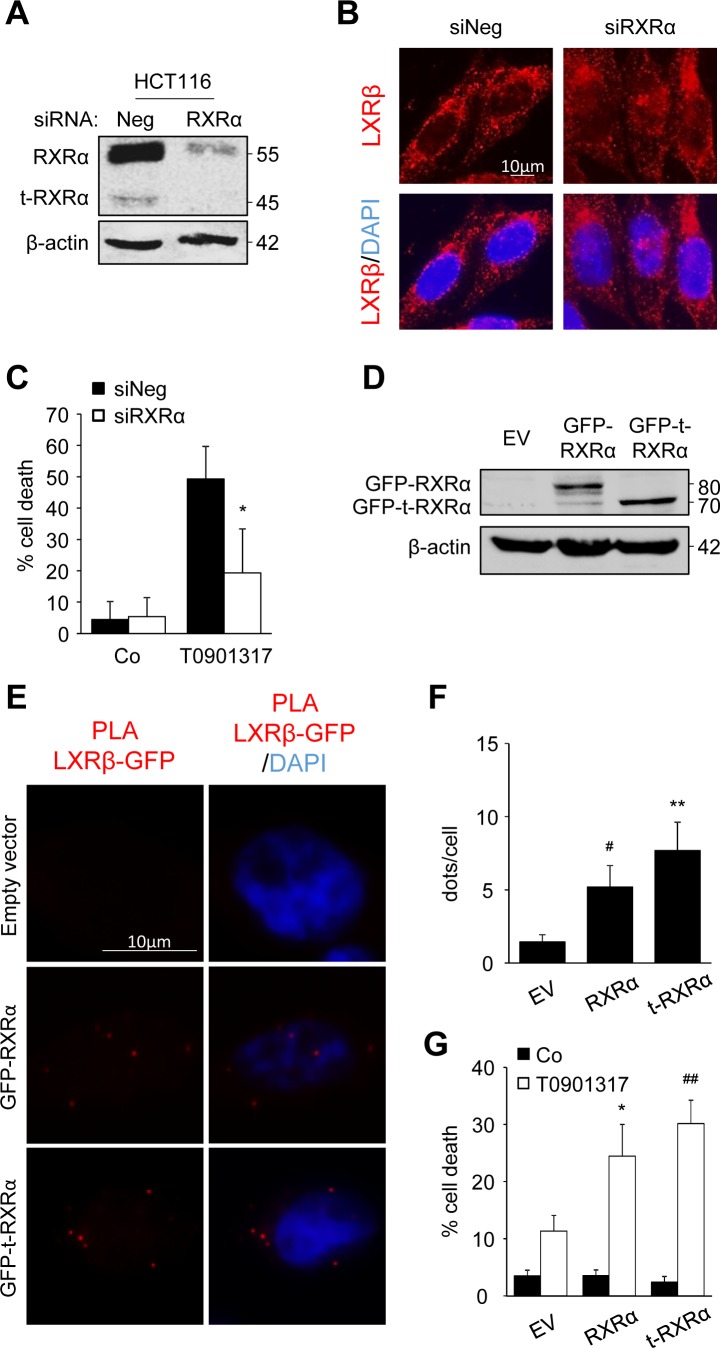
t-RXRα dictates LXRβ subcellular localization and colon cancer cell sensitivity **A.** - **C.** HCT116 cells were transiently transfected with negative siRNA or with siRNA targeting RXRα. **A.** Western blot analysis of RXRα and t-RXRα protein expression. β-Actin was used as a loading control. Numbers indicate molecular masses in kilodaltons. One representative experiment out of three. **B.** Immunofluorescence staining of LXRβ. Above: anti-LXRβ (red). Below: merge of LXRβ staining with DAPI (blue). **C.** Cell death determined by crystal violet coloration of transfected cells exposed to 20μM of T0901317 for 24 hours. Mean of three independent experiments ± s.d.. **D.** - **G.** SW620 cells were transiently transfected with empty vector (EV) or plasmid constructions containing RXRα or t-RXRα. **D.** Western blot analysis of GFP-RXRα and GFP-t-RXRα protein expression. β-Actin was used as a loading control. Numbers indicate molecular masses in kilodaltons. One representative experiment out of two. **E.** PLA between LXRβ and GFP-RXRα or GFP-t-RXRα or empty vector. Left: PLA LXRβ/GFP (red). Right: merge with DAPI (blue). **F.** Mean quantification of PLA interaction dots in three different experiments ± s.d.. **G.** Cell death determined by crystal violet coloration of transfected cells exposed to 20μM of T0901317 for 24 hours. Mean of three independent experiments ± s.d.. Statistics compare cells treated only with T0901317 with other treated conditions: * p < 0.05, **C.** or cells transfected with empty vector with cells transfected with other constructions: *p < 0.05, ^#^*p* < 0.01, **p < 0.005, ^##^*p* < 0.001 (**F.** and **G.**), using two tailed *t* test.

In order to prove the importance of t-RXRα, we first turned off RXRα and t-RXRα expression, using siRNA (Figure 4A). The decreased expression of RXRα and t-RXRα led to partial relocalization of LXRβ to the nucleus of HCT116 cells whereas beforehand it was mostly localized in the cytoplasm (Figure 4B). Moreover, this silencing turned T0901317-sensitive cells into T0901317-resistant cells (Figure 4C). In order to distinguish between the effects of the two forms of RXRα, we over-expressed RXRα or t-RXRα in SW620 cells, one of the most resistant cell lines with a low expression of t-RXRα (Figure 4D). First, a proximity ligation assay was used to visualize and localize the interaction with LXRβ. We noticed binding of LXRβ with the two forms of RXRα (Figure 4E and 4F). The interaction with t-RXRα seemed to occur in the cytoplasm, whereas the association with RXRα was found in both the nucleus and the cytoplasm (Supplementary Movies). Finally, the overexpression of RXRα or t-RXRα sensitized SW620 cells to T0901317 or 9-cisRA cytotoxicity (Figure 4G and supplementary Figure 3).

### A non-cancerous colon mucosa cell line is not sensitive to a treatment with an LXR agonist

**Figure 5:**
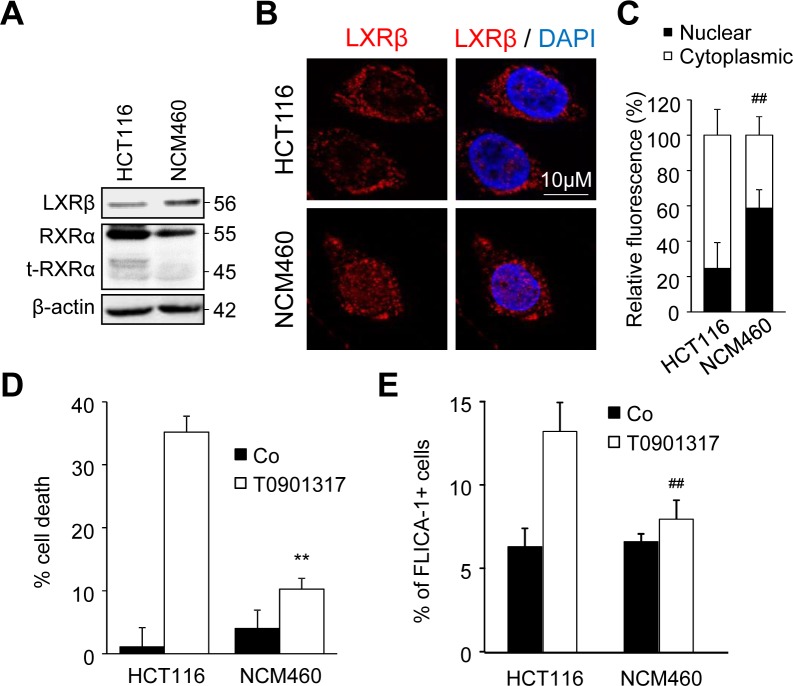
NCM460 cells are resistant to treatment with an LXR agonist **A.** Western blot analysis of LXRβ, RXRα and t-RXRα protein expression in HCT116 and NCM460 cell lines. β-Actin was used as a loading control. Numbers indicate molecular masses in kilodaltons. One representative experiments out of three. **B.** Representative images of immunofluorescence staining of LXRβ in HCT116 and NCM460 cells. Left: anti-LXRβ (red). Right: merge of LXRβ with DAPI (blue). **C.** Mean relative quantification of LXRβ fluorescence in the nucleus (black) and the cytoplasm (white) in three different experiments ± s.d.. **D.** - **E.** HCT116 or NCM460 cells were treated or not with 20μM of T0901317. **D.** Cell viability was determined by crystal violet coloration after 72 hours of treatment. **E.** Caspase-1 activation was determined with FLICA-1 staining after one hour of treatment. Data are the mean of three independent experiments ± s.d.. Statistics compare NCM460 cells with HCT116 cells in the same treated conditions: **p < 0.005, ^##^*p* < 0.001, **C.** to **E.** using two tailed *t* test.

In order to show that cytotoxicity of LXR ligands was specific to tumor cells, we used the normal derived colon mucosa cell line NCM640. We first showed that the amount of LXRβ expressed by NCM460 cells was similar to that expressed by HCT116 cells, whereas the expression of RXRα and t-RXRα was largely decreased even absent in the former (Figure 5A). Moreover, while LXRβ was mainly localized in the cytoplasm in HCT116 cells, it was mainly expressed in the nucleus of NCM460 cells (Figure 5B and 5C). We have previously reported that LXR agonists induced colon cancer cell death in a caspase-1-dependent manner [4]. T0901317 was indeed able to induce cell death (Figure 5D) and caspase-1 activation (Figure 5E) in HCT116 cells but not in NCM460 cells. Altogether, these results suggest that the absence of t-RXRα in normal colon cells leads to LXRβ localization in the nucleus and to a lesser extent makes these cells resistant to LXR ligands.

### LXR agonist targets colon cancer cells and spares normal colon mucosa cells from patients

**Figure 6:**
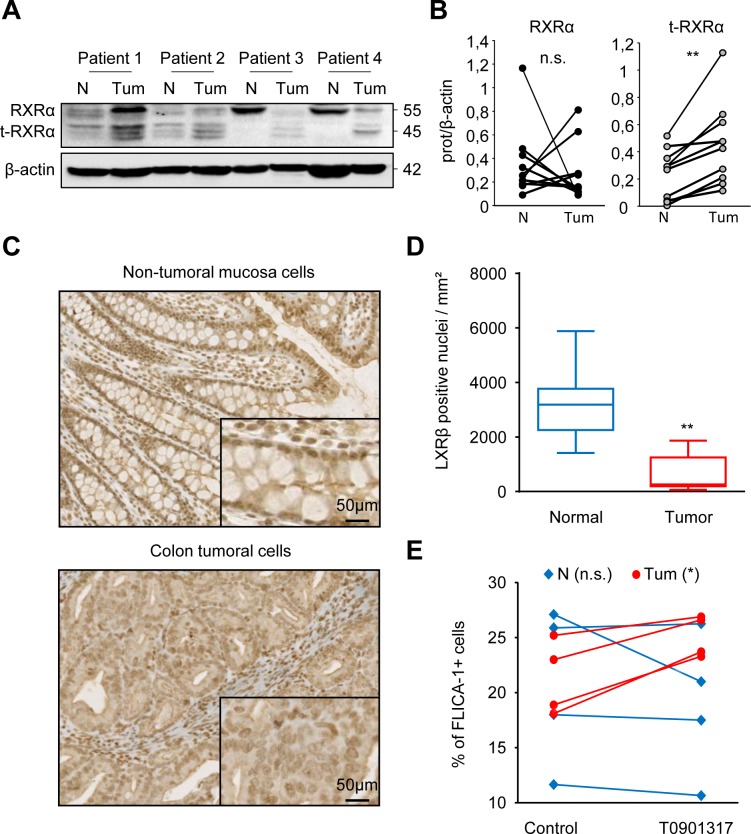
Normal colon mucosa cells from colon cancer patients are not sensitive to treatment with an LXR agonist **A.** Western blot analysis of RXRα and t-RXRα protein expression in Tumoral (Tum) or healthy peripheral Non-tumoral (N) tissues from colon cancer patients. β-Actin was used as a loading control. Numbers indicate molecular masses in kilodaltons. **B.** Mean of the quantification of the RXRα/β-actin (black) and the t-RXRα/ β-actin (grey) ratios in Tumoral or Non-Tumoral tissues from ten colon cancer patients. **C.** Immunohistochemical staining of LXRβ in non-tumoral colonic mucosa (upper panel) and tumoral tissue (lower panel) from human colon cancer patients. One representative patient out of ten. **D.** Mean relative quantification of the LXRβ staining in the nucleus in tumoral (red) or non-tumoral cells (blue) in ten different patients ± s.d.. **E.** FLICA-1 positive cells in tumoral (Tum- red circles) or Non-tumoral (N - blue diamonds) tissues of colon cancer patients treated *ex-vivo* with 20μM T0901317 or the vehicle (control) for one hour. Statistics compare tumors with Non-tumor samples: **p < 0.005, n.s., not significant (**B.** and **D.**) or T0901317-treated samples with untreated samples (control): *p < 0.05, n.s., not significant **E.**, using two tailed *t* test.

Samples of human colon tumors and paired adjacent healthy tissues were collected. We first showed that expression of RXRα and t-RXRα in normal tissues was different from that in tumor tissues (Figure 6A and 6B and Supplementary Figure 4). Secondly, we observed by immunohistochemistry that LXRβ was predominantly localized in the nucleus of normal colon epithelial cells whereas its localization in tumor cells was nucleo-cytoplasmic (Figure 6C and 6D and Supplementary Figure 5). Finally, samples of human colon tumors and adjacent healthy tissues were collected, dissociated and treated *ex-vivo* with T0901317. We observed that T0901317 activated caspase-1 in tumor samples but not in healthy tissues (Figure 6E). All these results suggest that tumor cells are characterized by a particular LXRβ localization, and that this localization makes cells sensitive to treatment with T0901317, which activates caspase-1. This cell-death cascade does not occur in normal epithelial cells.

## DISCUSSION

This study reports for the first time that LXRβ subcellular localization is responsible for the sensitivity of colon cancer cells to T0901317 cytotoxicity. The correlation between the cytoplasmic localization of LXRβ and the sensitivity of colon cancer cells to T0901317 cytotoxicity strengthens our previous work that described a non-genomic effect of LXRβ in these cells [1, 4]. We also described the importance of t-RXRα expression in these effects. Moreover, normal colon epithelial cells display predominantly nuclear expression of LXRβ, a lower t-RXRα level and are resistant to T0901317, thus highlighting that LXR ligands could be potential targets for therapy with lower side effects on healthy cells.

Zhou *et al*. identified the truncated form of RXRα, t-RXRα, in different cancer cell lines and in breast and liver primary tumors [9]. In accordance with this work, we also reported that t-RXRα was present in colon cancer samples but not in adjacent normal epithelial cells. This form of RXRα is generated by cleavage of the full length RXRα by calpain II and was shown to be responsible for high cancer cell proliferation *in vitro* and *in vivo* through phosphorylation and activation of Akt [9, 10] Here, we showed that cells with high expression of t-RXRα present a predominantly cytoplasmic LXRβ localization and a greater sensitivity to LXR ligand-induced cell death. Consequently, we propose here that treatment with LXR ligand could be an effective way to eradicate tumor cells with a high proliferation index.

Three non-exhaustive hypotheses are suggested by the literature to explain the different subcellular localization of LXRβ between normal epithelial cells and tumor cells. First, cancer pathogenesis is characterized by DNA mutations of genes. For example, LXRβ genotypes (rs2695121 - TC/CC) and (rs35463555 - GA/AA) were associated with the risk of developing gallbladder cancer [11]. Sequencing of LXRβ mRNA in HCT116 and NCM460 cells, which harbor distinct localizations, only showed a silent mutation that had no consequence on the amino acid sequence of the protein (not shown). Secondly, the nuclear localization of LXRβ could be explained by various amounts of endogenous ligands such as glucose or oxysterols [12, 13]. In our study, cells were cultured in different media with glucose concentrations ranging from 5.5mM to 11mM and there was no correlation between the medium glucose concentration and LXRβ subcellular localization. In mammals two main sources of oxysterols are available: exogenous oxysterols, provided by nutritional supply and endogenous oxysterols produced by enzymatic synthesis [13]. Greater quantities of oxysterols were shown to be produced in cancer cells than in adjacent normal breast tissue [14] and in colon cancer cell lines than in fibroblasts [15]. Further experiments should be done to clearly eliminate these hypotheses. Third, LXRβ can be sequestrated in the cytoplasm by a partner. One candidate is the truncated form of RXRα, t-RXRα which has been described as being located in the cytoplasm and as acting non-genomically on cancer cell growth and resistance [9]. Here, we provide evidence that t-RXRα and, to a lesser extent RXRα, bind with LXRβ and dictate 1) its subcellular localization, 2) colon cancer cell sensitivity and 3) heathy cell resistance towards LXR agonist cytotoxicity. In our study and in previous works that reported the cytoplasmic localization of t-RXRα [9], no experiments showed why this truncated form was cytoplasmic even though its NLS remained. One can speculate that another partner may be involved in the sequestration of the LXRβ/t-RXRα complex and further proteomic experiments will be needed to answer this question.

As observed with t-RXRα, the over-expression of RXRα in SW620 cells also triggers an interaction with endogenous LXRβ and an increase in LXR ligand cytotoxicity. This association seems to occur in the nucleus and the cytoplasm even though only a cytoplasmic interaction of t-RXRα with LXRβ was shown. The implication of RXRα overexpression in LXR-induced cell death may be partly explained by the cleavage of the transfected full length protein. However, we cannot exclude the possibility that RXRα plays a role in these effects.

Importantly, the clinical relevance of our work raises the hypothesis that LXRβ agonists could be a potential targeted therapy in colon cancer treatment. This is sustained by two main observations. First, LXRβ is specifically localized in the cytoplasm of human colon cancer cells whereas localization is predominantly nuclear in healthy mucosa cells. Second, human colon tumor cells but not normal epithelial cells are sensitive to treatment with an LXR ligand. These findings thus support the idea that LXRβ localization correlates with its ligand effects. Finally, immunohistological studies of the subcellular localization of LXRβ could be a potential companion biomarker of the efficacy of LXRβ agonists not only in colon cancer but also in other cancer models.

To conclude, the importance of LXR ligands in the treatment of malignancies such as cancer has been proved by many studies [1]. However, many questions still remain for whose working on the application of LXR ligands in cancer therapy, such as the profile expression of LXRs in clinical samples, the existence of other target cells or the adverse effects of LXR ligands [16]. This work will contribute to the better understanding of LXR ligand cytotoxicity (due to LXRβ subcellular localization) in colon cancer cells without affecting normal epithelial cells.

## MATERIALS AND METHODS

### Cell culture

The human colorectal carcinoma HCT116, colorectal adenocarcinoma HT29, HCT8, SW480, SW620, LoVo and SW48 cell lines were obtained from the American Type Culture Collection (ATCC). Some cell lines were grown in RPMI 1640 with ultraglutamine (Lonza, Levallois, France) (HCT116, HT29, HCT8, SW480) or in DMEM 4.5g/L glucose (Lonza) (SW620, LoVo, SW48) supplemented with 10% (vol/vol) fetal bovine serum (FBS; Lonza). The human normal mucosa cell line NCM460 was obtained from INCELL corporation LLC (San Antonio, TX) and was grown in M3Base medium (INCELL corporation LLC) supplemented with 10% FBS. All cell lines were grown in an atmosphere of 95% air and 5% CO_2_ at 37°C.

### Reagents

T0901317 was purchased from Bertin Pharma (Montigny le bretonneux, France), calpeptin from Sigma-Aldrich (Saint Quentin Fallavier, France) and 9cis-RA from Santa Cruz biotechnology (Heidelberg, Germany) and were dissolved in DMSO (Sigma-Aldrich).

### Cell death assay

Cells were seeded in 24-well plates at 50 000 cells/mL the day before treatment with the LXR agonist. After treatment, cells were washed twice with PBS and fixed with 100% ethanol for 30 minutes before crystal violet (Sigma-Aldrich) staining. Crystal violet was then suspended in 33% acetic acid and the OD was read at 590nm with a Wallac 2 spectophotometer (PerkinElmer, Courtaboeuf, France).

### Caspase activity

To assess caspase-1 activity, FAM-YVAD-FMK fluorescent probe (AbdSerotec, Kidlington, UK) binding to cleaved caspases was used according to the manufacturer's instructions. Briefly, 250 000 cells were concomitantly incubated with the probes and LXR agonist for 1 hour and then washed twice in apoptosis buffer before flow cytometry analysis. For measurements in tumor and healthy tissues from colon cancer patients, samples were dissociated using collagenase (C0130) and DNAse (D5025) (Sigma-Aldrich) treatment at 37°C before incubation with probes.

### Immunoprecipitation

Cells (50 × 10^6^) were lysed in 1 mL lysis buffer (20 mM Tris [pH 7.5], 14.5 mM KCl, 5mM MgCl_2_, 1mM EDTA, 1mM EGTA, 1% CHAPS and complete protease inhibitor mixture (Roche diagnotics, Meylan, France)) for 30 minutes on ice. After centrifugation at 14 000g at 4°C for 30 minutes, supernatants were pre-cleared for 1 hour at 4°C in the presence of 30 μL of mixed Sepharose 6B (6B100, Sigma Aldrich) and protein G (17-0618-01, GE healthcare, Velizy-Villacoublay, France). After centrifugation at 1000g for 3 minutes the supernatant was incubated with 2μg/mL of anti-LXRβ antibody (PP-K8917, R&D sytems, Abingdon, UK) at 4°C for 20 hours and during the last hour with 40 μL of mixed Sepharose. The precipitates were washed 4 times in lysis buffer and analyzed by immunoblotting.

### Western blotting

Whole-cell lysates were prepared as described previously [4], by lysing the cells in boiling buffer (1% SDS, 1 mM sodium vanadate, 10 mM Tris [pH 7.4]) in the presence of complete protease inhibitor mixture (Roche diagnostics). The viscosity of the samples was reduced by sonication. Whole-cell lysates or immunoprecipitation samples were separated by sodium dodecyl sulfate–polyacrylamide gel electrophoresis (SDS-PAGE), and electroblotted onto a nitrocellulose membrane (GE Healthcare). After incubation for one hour at RT with 5% nonfat milk in Tris-buffered saline (TBS)–0.1% Tween-20, membranes were incubated overnight with the primary antibody diluted in TBS-milk-Tween, washed, incubated with the secondary antibody for 30 minutes at RT, and washed again before analysis with a luminol detection kit (Santa Cruz biotechnology) and chemidoc analyser (Bio-Rad, Marnes-la-coquette, France). Relative quantification was performed using ImageLab software. The following mouse mAbs were used: anti–β-actin (A1978) from Sigma-Aldrich, anti- LXRβ (PP-K8917) from R&D sytems. We also used rabbit pAbs anti-RXRα (ΔN197 - Santa Cruz biotechnology) and anti-GFP (PA1-980A – Fisher scientific, Illkirch, France). Secondary Abs HRP-conjugated polyclonal goat anti-mouse and swine anti-rabbit immunoglobulins (Jackson ImmunoResearch, Suffolk, UK) were also used.

### Transient transfections

Human SW620 cells were transiently transfected for 24 hours with expression plasmids using the JetPRIME reagent (PolyPlus transfection, Illkirch, France), according to the manufacturer's instructions. The following plasmids were used: pcDNA6.2/C-EmGFP TOPO plasmid (Life technologies) containing the 1380bp human RXRα coding sequence or the t- RXRα sequence obtained by deletion of the 3′-end 230bp.

HCT116 cells were transfected with the INTERFERin™ transfection reagent (Polyplus transfection) according to the manufacturer's instructions. The following siRNA (Life Technologies) were used: rxrα (s12385) or control (AM4611).

### Immunofluorescence (IF) and in situ Proximity Ligation Assay (PLA)

Cells (150 000) were seeded in 12 well-dishes containing a cover glass (631-0150, VWR, Fontenay-sous-bois, France) which was pretreated for 10 minutes with Poly-L-Lysin (P4707, Sigma Aldrich). The following day cells were treated or not with LXR agonists for the indicated times. Cells were washed, fixed with 4% PFA at 4°C for 10 minutes and permeabilized using a PBS, 3% BSA, 0.2% Saponin (47036, Sigma Aldrich) buffer for 20 minutes at RT. Samples were incubated overnight at 4°C with primary antibodies.

For IF experiments, cells were washed twice, and incubated with secondary Alexa568 coupled anti-rabbit (Life technologies, Saint Aubin, France) for 30 minutes at RT. For PLA experiments, after excess primary antibodies had been washed off, cells were incubated with the appropriate probes (Sigma Aldrich) for one hour at 37°C and washed twice. Probes were then ligated for 30 minutes at 37°C, washed two times in Buffer A and amplified using the manufacturer's polymerase for 100 minutes at 37°C in the dark.

For both experiments, cover glasses were mounted on a drop of Mounting Medium containing Dapi (Duo82040, Sigma Aldrich) for 15 minutes in the dark on a microscopy slide (045796, Dutscher, Brumath, france). Slides were imaged using a CDD equipped upright microscope (Zeiss, Marly le Roi, France) and 63x, 1.4NA objective. To evaluate the mean fluorescence of LXRβ in the nucleus and the cytoplasm, ImageJ software was used. The integrative density of LXRβ staining corresponding to the nucleus was calculated in the area matching with DAPI staining. For the cytoplasm, the integrative density of nuclear LXRβ staining was subtracted from the integrative density of LXRβ staining for the whole cell. Then percentages of the distribution of LXRβ in the nucleus and the cytoplasm was calculated. The means +/− s.d. were calculated from about 10 images (containing at least 15 cells) obtained from three independent experiments.

The following antibodies were used for IF and PLA: rabbit anti LXRβ (1/250, ab106473, Abcam, Paris, France), mouse anti-GFP B-2 (1/50, sc9996, Santa Cruz biotechnology), anti-rabbit PLUS probe (1/5, Duo92002, Sigma-Aldrich), anti-mouse MINUS probe (1/5, Duo92004, Sigma-Aldrich), goat anti-rabbit Alexa568 (1/1000, A11036, Life technologies).

### Immunohistochemistry

Formalin-fixed, paraffin-embedded tumor sections were labelled using the ultraVIEW DAB system (Ventana, Basel, Switzerland). Briefly, antigen retrieval was carried out by heating slides for 90 minutes at 95°C in 1 mmol/L EDTA at pH 7.8. Slides were then incubated with LXRβ mAb (ab24361 (Abcam) at a 1/20 dilution for 32 min at 37°C. The stained arrays were counterstained with hematoxylin and mounted in Aquamount (Dako, Les Ulis, France). Labeling was detected using a Ventana Benchmark XT automat (Ventana).

Slides were then digitized using Hamamatsu NanoZoomer 2.0HT and NDP Scan software (Hamamatsu Photonics, Massy, France). Nucleus detection and quantification were performed using TissueStudio 3.6.1 software (Definiens, Munich, Germany).

### Ethical issues

Patients provided their written informed consent for the use of samples from their tumors for future investigations at the time of the diagnosis and experiments were approved by the local ethics committee (Institutional Review Board of the Centre Georges Francois Leclerc).

### Statistical analyses

*In vitro* results are shown as means ± s.d. and comparisons of datasets were performed using unpaired Student's *t* test (test group compared with control group). For human experiments, a paired *t* test was used to compare samples from the same patients before and after one hour of T0901317 treatment or to compare tumor samples with non-tumor samples. We performed statistical calculations with GraphPad Prism 5. All *P* values were two tailed.

## SUPPLEMENTARY MATERIAL FIGURES AND MOVIES







## References

[1] Rébé C, Derangère V, Ghiringhelli F (2015). Transcriptional and non-transcriptional roles of LXRs in cancer cells. Receptor Cin Invest.

[2] Guo D, Reinitz F, Youssef M, Hong C, Nathanson D, Akhavan D, Kuga D, Amzajerdi AN, Soto H, Zhu S, Babic I, Tanaka K, Dang J, Iwanami A, Gini B, Dejesus J (2011). An LXR agonist promotes glioblastoma cell death through inhibition of an EGFR/AKT/SREBP-1/LDLR-dependent pathway. Cancer discovery.

[3] Pommier AJ, Alves G, Viennois E, Bernard S, Communal Y, Sion B, Marceau G, Damon C, Mouzat K, Caira F, Baron S, Lobaccaro JM (2010). Liver X Receptor activation downregulates AKT survival signaling in lipid rafts and induces apoptosis of prostate cancer cells. Oncogene.

[4] Derangere V, Chevriaux A, Courtaut F, Bruchard M, Berger H, Chalmin F, Causse SZ, Limagne E, Vegran F, Ladoire S, Simon B, Boireau W, Hichami A, Apetoh L, Mignot G, Ghiringhelli F (2014). Liver X receptor beta activation induces pyroptosis of human and murine colon cancer cells. Cell death and differentiation.

[5] Rébé C, Derangère V, Ghiringhelli F (2015). Induction of pyroptosis by LXRβ in colon cancer cells. Molecular & Cellular Oncology.

[6] Prufer K, Boudreaux J (2007). Nuclear localization of liver X receptor alpha and beta is differentially regulated. Journal of cellular biochemistry.

[7] Miller A, Crumbley C, Prufer K (2009). The N-terminal nuclear localization sequences of liver X receptors alpha and beta bind to importin alpha and are essential for both nuclear import and transactivating functions. The international journal of biochemistry & cell biology.

[8] Edwards PA, Kast HR, Anisfeld AM (2002). BAREing it all: the adoption of LXR and FXR and their roles in lipid homeostasis. Journal of lipid research.

[9] Zhou H, Liu W, Su Y, Wei Z, Liu J, Kolluri SK, Wu H, Cao Y, Chen J, Wu Y, Yan T, Cao X, Gao W, Molotkov A, Jiang F, Li WG (2010). NSAID sulindac and its analog bind RXRalpha and inhibit RXRalpha-dependent AKT signaling. Cancer cell.

[10] Gao W, Liu J, Hu M, Huang M, Cai S, Zeng Z, Lin B, Cao X, Chen J, Zeng JZ, Zhou H, Zhang XK (2013). Regulation of proteolytic cleavage of retinoid X receptor-alpha by GSK-3beta. Carcinogenesis.

[11] Sharma KL, Misra S, Kumar A, Mittal B (2013). Association of liver X receptors (LXRs) genetic variants to gallbladder cancer susceptibility. Tumour Biol.

[12] Helleboid-Chapman A, Helleboid S, Jakel H, Timmerman C, Sergheraert C, Pattou F, Fruchart-Najib J, Fruchart JC (2006). Glucose regulates LXRalpha subcellular localization and function in rat pancreatic beta-cells. Cell Res.

[13] Viennois E, Mouzat K, Dufour J, Morel L, Lobaccaro JM, Baron S (2012). Selective liver X receptor modulators (SLiMs): what use in human health?. Molecular and cellular endocrinology.

[14] Wu Q, Ishikawa T, Sirianni R, Tang H, McDonald JG, Yuhanna IS, Thompson B, Girard L, Mineo C, Brekken RA, Umetani M, Euhus DM, Xie Y, Shaul PW (2013). 27-Hydroxycholesterol promotes cell-autonomous, ER-positive breast cancer growth. Cell reports.

[15] Roberg-Larsen H, Strand MF, Grimsmo A, Olsen PA, Dembinski JL, Rise F, Lundanes E, Greibrokk T, Krauss S, Wilson SR (2012). High sensitivity measurements of active oxysterols with automated filtration/filter backflush-solid phase extraction-liquid chromatography-mass spectrometry. Journal of chromatography A.

[16] Lin CY, Gustafsson JA (2015). Targeting liver X receptors in cancer therapeutics. Nature reviews Cancer.

