# Supplementary material for: Liver X Receptor ligand cytotoxicity in colon cancer cells and not in normal colon epithelial cells depends on LXRβ subcellular localization
Source: Oncotarget. 2015 Sep 22;6(29):26651–62. doi: 10.18632/oncotarget.5791 (PMC4694942; doi:10.18632/oncotarget.5791)
Supplement: Supplementary file 1 [file oncotarget-06-26651-s001.pdf]

Liver X Receptor ligand cytotoxicity in colon cancer cells and not in normal colon epithelial cells depends on LXRβ subcellular localization

Supplementary Material

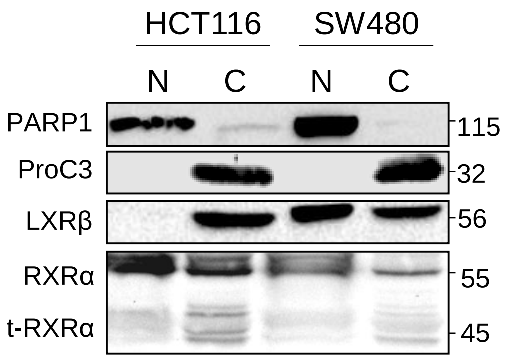

Supplementary Figure 1: Subcellular localization of LXRβ, RXRα and t-RXRα. Western blot analysis of LXRβ, RXRα and t-RXRα protein expression in HCT116 and SW480 human colon cancer cell lines. PARP1 and Procaspase-3 were used as a nuclear or cytoplasmic fraction control respectively. Numbers indicate molecular masses in kilodaltons. N = Nuclear fraction and C = cytoplasmic fraction.

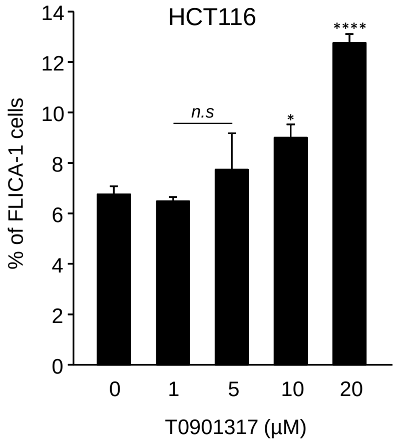

Supplementary Figure 2: LXR ligand induces caspase-1 activation. HCT116 cells were treated with indicated concentrations of T0901317 for 1 hour. Caspase-1 activation was determined using FLICA-1. Mean of three independent experiments ± s.d.. Statistics compare cells treated with T0901317 with untreated cells: * $p<0.01$, **** $p<0.0001$, n.s., not significant using two tailed t test.

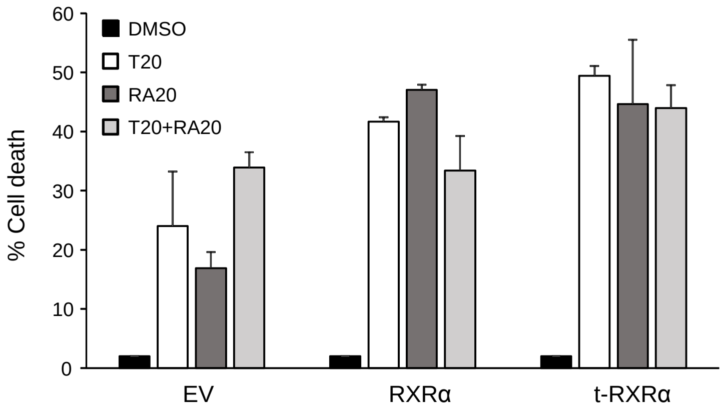

Supplementary Figure 3: Effects of RXRα and t-RXRα overexpression on LXR and RXR ligand toxicities. SW620 cells were transiently transfected with empty vector (EV) or plasmids coding for RXRα or t-RXRα. Cells were then treated either with 20µM of T0901317 (T20-white bars) or 20µM of 9-cisRA (RA20-dark grey bars) or both (T20+RA20-ligth grey bars) or DMSO (black bars). One representative experiment out of three.

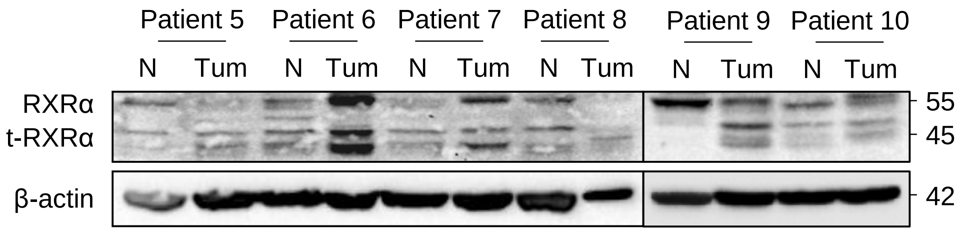

Supplementary Figure 4: RXRα and t-RXRα expression in normal and tumor samples from colon cancer patients. Western blot analysis of RXRα and t-RXRα protein expression in Tumoral (Tum) or healthy peripheral Non-Tumoral (N) tissues from colon cancer patients. β-Actin was used as a loading control. Numbers indicate molecular masses in kilodaltons.

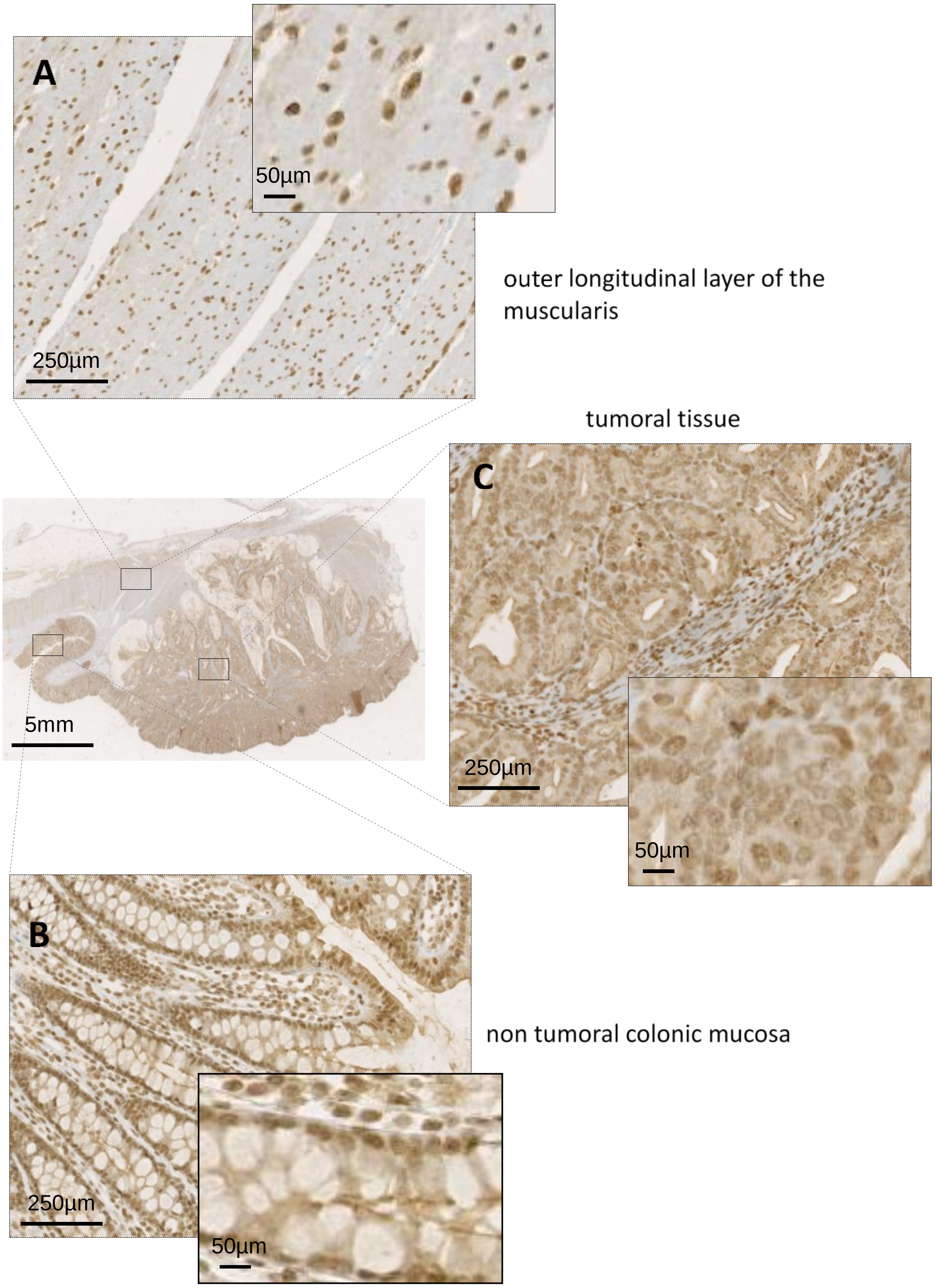

Supplementary Figure 5: LXRβ localization in normal and tumor samples from colon cancer patients. Example of immunohistochemical staining of LXRβ on human colon cancer patient samples. Localization of the different areas studied = upper: muscularis; middle = tumoral tissue; lower = non-tumoral colonic mucosa. One representative patient out of ten.

SUPPLEMENTARY MOVIES: Subcellular localization of LXRβ-RXRα or LXRβ-t-RXRα interactions. PLA between LXRβ and GFP-RXRα or GFP-t-RXRα. The Z-stacks from the image in Figure 4e were compiled into a 3-dimensional image and rotated in a movie.

SUPPLEMENTARY MATERIAL AND METHODS

Nuclear and cytoplasmic fraction isolation

Cells were incubated in hypotonic buffer (20 mM Hepes pH7.5, 10mM KCl, 1.5mM $MgCl_2$, 1mM EDTA, 1mM EGTA, 250mM sucrose) in the presence of complete protease inhibitor mixture for 30 min. on ice. Then cells were passed through a 26G needle and centrifuged at 1000g for 10 min. at 4°C. The pellet correspond to the nuclear fraction and the supernatant to the cytoplasmic fraction. Lysates were then analysed by western blotting. Anti-Caspase-3 (8G10) from Cell Signaling (Saint Quentin Yvelines) and anti-PARP-1 from Santa cruz biotechnology antibodies were used.
